# Network-based method for regions with statistically frequent interchromosomal interactions at single-cell resolution

**DOI:** 10.1186/s12859-020-03689-x

**Published:** 2020-09-30

**Authors:** Chanaka Bulathsinghalage, Lu Liu

**Affiliations:** North Dakota State University, 1340 Administration Ave, Fargo, 58102 USA

**Keywords:** Single-cell Hi-C, Interchromosomal interactions, Networks, Statistically frequent

## Abstract

**Background:**

Chromosome conformation capture-based methods, especially Hi-C, enable scientists to detect genome-wide chromatin interactions and study the spatial organization of chromatin, which plays important roles in gene expression regulation, DNA replication and repair etc. Thus, developing computational methods to unravel patterns behind the data becomes critical. Existing computational methods focus on intrachromosomal interactions and ignore interchromosomal interactions partly because there is no prior knowledge for interchromosomal interactions and the frequency of interchromosomal interactions is much lower while the search space is much larger. With the development of single-cell technologies, the advent of single-cell Hi-C makes interrogating the spatial structure of chromatin at single-cell resolution possible. It also brings a new type of frequency information, the number of single cells with chromatin interactions between two disjoint chromosome regions.

**Results:**

Considering the lack of computational methods on interchromosomal interactions and the unsurprisingly frequent intrachromosomal interactions along the diagonal of a chromatin contact map, we propose a computational method dedicated to analyzing interchromosomal interactions of single-cell Hi-C with this new frequency information. To the best of our knowledge, our proposed tool is the first to identify regions with statistically frequent interchromosomal interactions at single-cell resolution. We demonstrate that the tool utilizing networks and binomial statistical tests can identify interesting structural regions through visualization, comparison and enrichment analysis and it also supports different configurations to provide users with flexibility.

**Conclusions:**

It will be a useful tool for analyzing single-cell Hi-C interchromosomal interactions.

## Background

Stretching the DNA in a human cell, it would be about two meters long, but how can it fit into a tiny space of about 6 microns across? DNA of cells of different tissues (e.g. neural cells and heart cells) are essentially the same, but why do these cells function disparately and what factors turn the genes’ on and off and result in the disparities? To gain insights into these questions, advances in chromosome conformation capture-based technologies have provided researchers a great opportunity to study the higher-order spatial organization of chromatin. A popular method is chromosome conformation capture with high-throughput sequencing (Hi-C), in which genomes are cross-linked with formaldehyde, fragmented with enzymes, randomly ligated in proximity and finally sequenced by next-generation sequencing platforms. After raw reads are processed by bioinformatics pipelines, a genome-wide contact map of a collection of cells is generated and it reveals intrachromosomal interactions and interchromosomal interactions. Intrachromosomal interactions refer to the valid ligations between DNA fragments of the same chromosome and interchromosomal interactions refer to the valid ligations between DNA fragments of different chromosomes. Intrachromosomal interactions are the majority of chromatin interactions in Hi-C experiments and their interaction frequencies are genomic distance dependent [1]. Interchromosomal interactions are two orders of magnitude weaker than intrachromosomal interactions [2] and interchromosomal interactions contain a higher proportion of noise than intrachromosomal interactions [3].

As the popularity of the Hi-C approach grows, large amounts of data have been generated and significant endeavors are devoted to developing computational methods and tools. These computational methods and tools can be coarsely divided into two categories, Hi-C data processing and downstream analysis. For the first category, there are some existing tools used to generate valid chromatin interactions from raw sequencing reads [4–12]. They follow similar processing steps and may adopt different sequence alignment strategies (pre-truncation, iterative and trimming), filtering criteria (read-level, read-pair level, strand and distance) and normalization methods (explicit-factor correction, matrix balancing and joint correction). Besides, there are some computational tools to exam the quality of Hi-C data by measuring the reproducibility of Hi-C replicates [10, 13–15]. For the second category, there are several major analysis tasks to gain insights into the spatial structure and function of chromatin. A/B compartments which correspond to open and closed chromatin can be identified by using Principle Component Analysis on transformed chromatin contact maps [16]. Megabase-sized Topologically Associating Domains (TADs) can be discovered by using a Hidden Markov Model with a directionality index [17]. There are other methods available to detect TADs [18–23]. As TADs are defined as continuous chromosomal loci, these methods only take intrachromosomal interactions into consideration. Statistically significant long-range chromatin interactions are extracted from Hi-C data. As there is no prior knowledge about interchromosomal interactions, computational methods focus on intrachromosomal interactions because the frequency of interactions between two intrachromosomal loci heavily depends on the genomic distance between the loci. Some methods identify statistically significant chromatin interactions by fitting the frequencies of intrachromosomal interactions with certain distributions, such as power-law [16], double-exponential [24] and negative binomial [25]. Instead of assuming a certain distribution, a nonparametric method [26] identifies statistically significant chromatin interactions by estimating the genomic distance-dependence relationship with splines. Furthermore, there is a method [19] extracting significant chromatin interactions as calling peaks in a chromatin contact map within the surrounding two-dimensional region. Hi-C data are also used to construct three-dimensional models of chromatin structure. Some methods [24, 27–33] try to learn a consensus chromatin structure of a collection of cells. Some methods [34–39] are intended to learn a set of chromatin structures representative of the observed chromatin interaction data. Besides the above downstream analysis tasks, there are some computational methods to carry out differential analysis on Hi-C data [40, 41] and multiple two-dimensional visualization tools exist [42–45]. For a comprehensive list of computational tools on Hi-C data, please check out the Omictools website [46] on high-throughput chromosome conformation capture data analysis software tools.

There are substantial computational methods and tools for downstream analysis of Hi-C data, however, most of them focus on intrachromosomal interactions and little attention is paid to interchromosomal interactions. Partly because there is no prior knowledge such as the strong genomic distance-dependence relationship between frequency of intrachromosomal interactions and the genomic distance. In addition, the frequency of the interchromosomal interactions is much lower than intrachromosomal interactions while their search space is much larger (bin pairs across chromosomes VS bin pairs within chromosomes). To the best of our knowledge, there are few computational studies that are dedicated to bulk Hi-C interchromosomal interactions. One study presents an investigation on human and mouse interchromosomal contacts and provides insights into mammalian chromatin organization [17]. A recent work develops a computational method based on an autoencoder and a multilayer perceptron classifier to impute high-resolution interchromosomal interactions [47]. Another paper presents two computational methods to estimate the transcription factors enriched in the interchromosomal interactions in yeast [48].

With the development of single-cell technologies, some single-cell Hi-C (scHi-C) approaches [49–51] are invented and therefore we can examine chromatin interactions at single-cell resolution. They also bring a new type of frequency information, the number of single cells with chromatin interactions between two disjoint chromosome regions. Generally these chromosome regions are defined by dividing chromosomes into equal-sized bins according to a resolution specified by users. Considering the lack of computational methods on interchromosomal interactions and the obvious pattern of intrachromosomal interactions along the diagonal of a chromatin contact map, we propose a computational method dedicated to analyzing interchromosomal interactions of single-cell Hi-C with this new frequency information. The fundamental difference between our research and previous research on interchromosomal interactions is our research is based on the new frequency information observed from each cell among all cells profiled. Since a bulk Hi-C experiment pools cells together at the very beginning so it can’t discern whether a chromosomal interaction is shared by single cells or not. Therefore, computational methods on bulk Hi-C experiments don’t consider the new frequency information at single-cell level, which is not available in bulk Hi-C experiments. In addition, when dealing with frequent interchromosomal interactions our method takes multiple contact maps as its inputs while computational methods on bulk Hi-C take one contact map as their inputs. What is more, to the best of our knowledge there is no tool available for frequent interchromosomal interactions. Specifically, we develop a computational tool to identify regions with statistically frequent interchromosomal interactions and make it accessible to the public. We believe that the regions associated with statistically frequent interchromosomal interactions under the single-cell context may be helpful for new hypotheses and functionally important therefore deserve more attention. Finally, frequent pattern mining is a longstanding topic in data mining research [52].

Our contributions may be stated as follows:

- We propose a computational method to identify regions associated with statistically frequent interchromosomal interactions at single-cell resolution.
- To the best of our knowledge, we are the first to implement a tool to serve the purpose and make it open to the public. To accommodate different scHi-C experiments, the tool is flexible on configurations.
- We demonstrate that using our proposed tool on two real scHi-C data sets, it can identify interesting structural regions.

The rest of paper is organized as follows. The “Method” delineates our proposed method in detail. The “Data” introduces two scHi-C data sets as our inputs. The “Results and discussion” demonstrates that our proposed tool’s usability on identifying interesting regions and flexibility of configurations. The “Conclusion” sections concludes that the tool will be useful for analyzing scHi-C interchromosomal interactions.

## Method

**Fig. 1:**
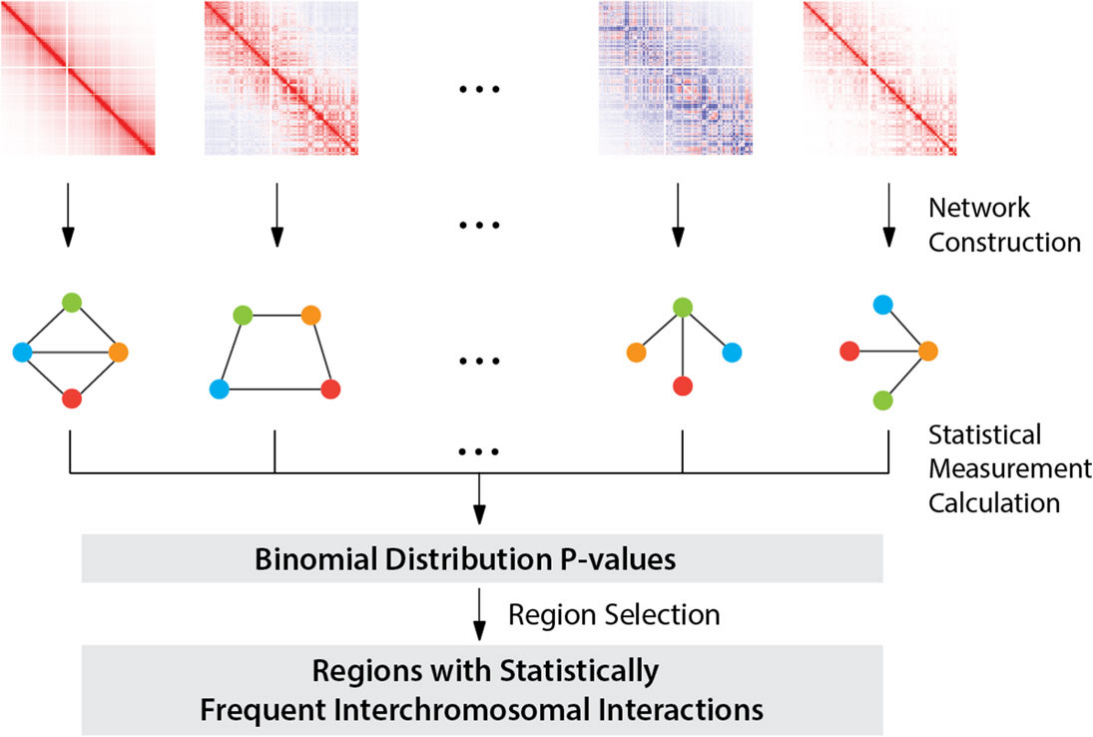
Workflow of the proposed method based on networks and statistical tests

In Fig. 1, the workflow of our proposed tool is illustrated and it includes three steps, network construction, statistical measurement calculation and region selection. The inputs of our tool are chromatin interactions of single cells, which are represented in heatmaps and can be easily generated with scHi-C processing pipelines such as NueProcess [53]. The outputs of our tool are identified regions, whose interchromosomal interactions are statistically frequent, along with frequencies and *p*-values. They are provided to help users refine identified regions with some frequency or *p*-value cutoff.

First, we construct a network by using interchromosomal interactions for each cell respectively. Due to low read coverages of scHi-C experiments and the more complex chromosomal structures of larger mammalian genomes, i.e. homo sapiens and mus musculus, chromosomes are divided into equal-sized bins to accumulate sufficient signals. Each bin is represented as a node with an index, and if there is an interchromosomal interaction whose two ends fall within two bins then the corresponding two nodes are connected with an edge. Instead of counting the number of interchromosomal interactions between bins, we are more concerned about their presence or absence because of the scarcity and variability of interchromosomal interactions in single cells. Therefore, an unweighted network is constructed for each cell.

Second, we develop a measurement to quantify how statistically frequent for an edge to be detected among single cells. To avoid an overestimation of this measurement and therefore reduce false positives, we first remove nodes without any intrachromosomal and interchromosomal interactions among all cells to narrow down the search space of edges, which originally is all node pairs of different chromosomes. Assume the number of edges in the edge search space is *M*, the number of single cells is *N*, and the number of interchromosomal interactions for cell *i* is represented as *n*_*i*_. Then $\frac {n_{i}}{M}$ represents the probability for cell *i* to have an edge between two nodes of different chromosomes. If a given edge is observed in *t* cells, we can use the following equations to calculate its *p*-value.

1 $$ p\text{-}value=\sum_{i=t}^{N}{N\choose i}p^{i}(1-p)^{N-i}   $$

2 $$ p=func\left(\frac{n_{1}}{M}, \frac{n_{2}}{M}, \cdots, \frac{n_{N-1}}{M}, \frac{n_{N}}{M}\right)   $$

3 $$ func\in\{max, mean, min\}   $$

Similar to previous research [27, 54, 55], in Eq. 1 the binomial distribution is applied to estimate the *p*-value that reflects how likely it is for an edge to be observed in at least a given number of cells among all single cells. The rationality behind the selection of the binomial distribution is assuming whether there is an edge between two nodes of different chromosomes is a Bernoulli trial, the binomial distribution can capture edges that appear so frequent in multiple single cells that they reach statistical significance among all single cells. These frequent edges can only be detected in scHi-C experiments instead of bulk Hi-C experiments because subtle single-cell level information is pooled in bulk Hi-C experiments. Equation 2 is used to quantify the probability of an edge with all cells considered, which is determined by a function in Eq. 3. Users can configure the selection of these functions through a parameter. For scHi-C experiments with larger genomes or low sequencing depths, it is recommended to use *max* to select regions with highly statistically frequent interchromosomal interactions; therefore fewer regions would be selected. To the contrary, *min* is applied to select more regions. For scHi-C experiments with smaller genomes or high sequencing depths, *min* increases the odds for some regions to be selected while *max* may find nothing. *mean* is a balance between *max* and *min*, so the number of identified regions falls between them.

At last, *p*-values are adjusted by the Bonferroni correction and a user provided *p*-value cutoff, e.g. 0.05, is applied to select regions associated with statistically frequent interchromosomal interactions.

## Data

To demonstrate that our proposed tool can be used to identify interesting structural regions, we use data from two existing scHi-C studies as our input data sets.

The first study [56] investigated the cell-cycle dynamics of chromosomal organization at single-cell resolution. The authors processed single *F*_1_ hybrid 129 × Castaneus mouse embryonic stem cells (mESCs) grown in 2i media using 1.5 million reads per cell on average. They analyzed 1,171 cells with fluorescence-activiated cell sorting, which labeled these cells to different cell-cycle phases based on levels of the DNA replication marker geminin and DNA content. Among them, 280 cells with a prefix of 1CDX1 were labeled as G1 phase; 303 cells with a prefix of 1CDX2 were labeled as Early-S phase; 262 cells with a prefix of 1CDX3 were labeled as Mid-S phase; 326 cells with a prefix of 1CDX4 were labeled as Late-S phase. We treat cells of different cell-cycle phases separately and feed them as inputs of our tool respectively. Therefore we identify regions with statistically frequent interchromosomal interactions for different cell-cycle phases.

The second one [50] developed a single-nucleus Hi-C protocol which provides >10-fold more contacts per cell than the previous method [49] to investigate chromatin organization at oocyte-to-zygote transition in mice. There are 40 transcriptionally active oocytes labeled as non-surrounded nucleolus (NSN), 76 transcriptionally inactive oocytes labeled as surrounded nucleolus (SN), 30 maternal nuclei from zygotes and 24 paternal nuclei from zygotes. Maternal and paternal nuclei are extracted from predominantly G1 phase zygotes.

## Results and discussion

Both data sets have single cells/nuclei of four conditions, therefore we run the proposed tool on single cells/nuclei of each condition respectively. Since the genomes used in the two experiments are large and sequencing read coverages are low, to accumulate sufficient interchromosomal interactions in a bin, we set the bin size to 500 kilobases (kb), which is also used in other existing studies [55, 57]. We first show that our tool can identify regions with statistically frequent interchromosomal interactions, then demonstrate that our tool is flexible to different configurations, which support sliding windows for region diversity, different functions to estimate the probability of having an edge between two nodes thereby providing adaptability of identified regions, and a configuration of different bin sizes e.g. 500kb VS 1 megabases (Mb).

### Usability of identifying interesting regions

To demonstrate the usability of our proposed method, we first display identified regions in visualization, then compare the identified regions and at last carry out enrichment analysis with other genomics features such as CTCF binding sites and enhancers etc.

#### Identification of statistically frequent regions

**Fig. 2:**
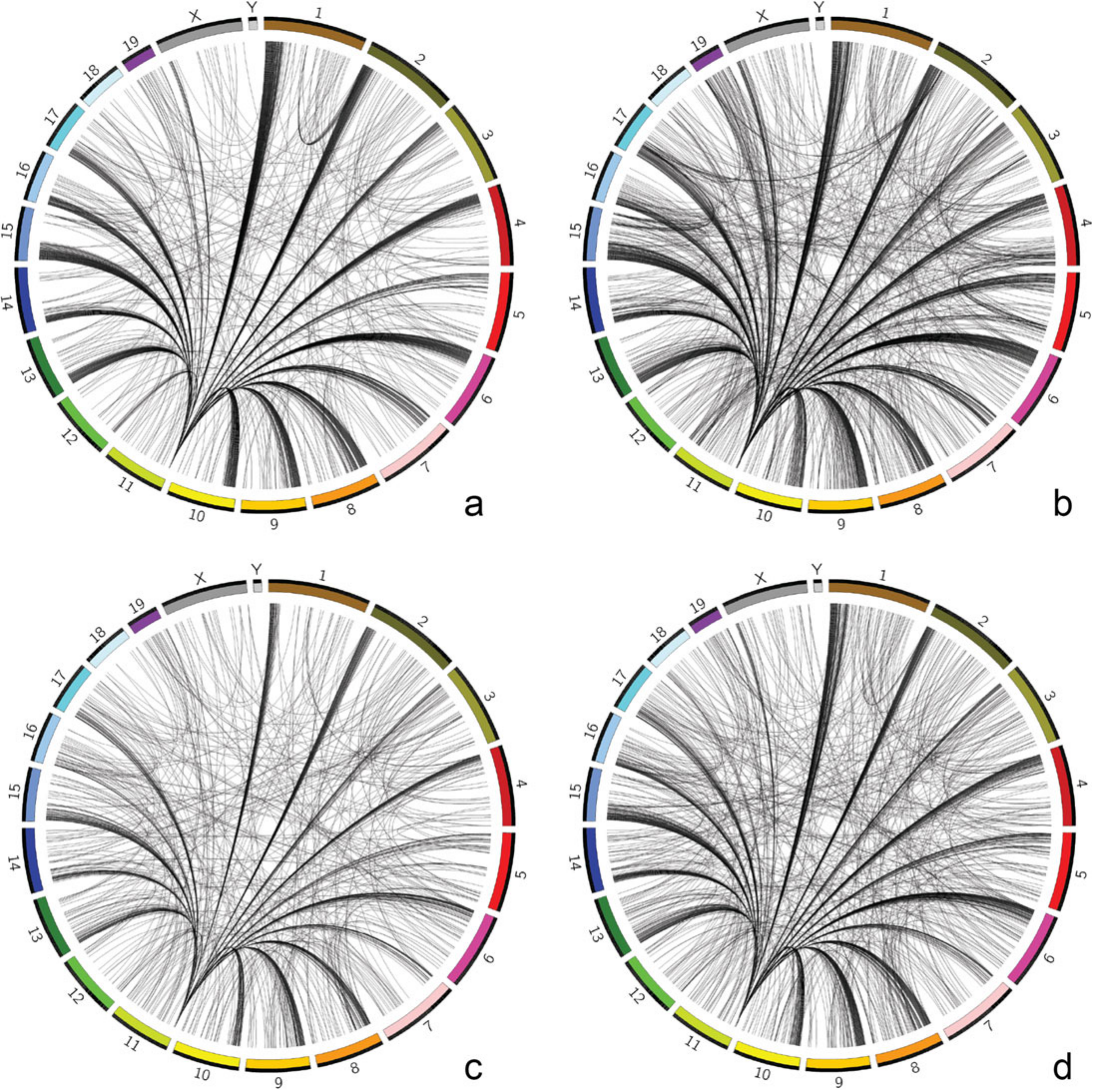
Identified regions of the cell-cycle data set. Visualizing genome-wide identified regions and their interchromosomal interactions of the cell-cycle data set with an adjusted *p*-value cutoff of 0.05 in Circos plots. **a** single cells of G1 phase; **b** single cells of Early-S phase; **c** single cells of Mid-S phase; **d** single cells of Late-S phase

In Fig. 2, identified regions associated with statistically frequent interchromosomal interactions among single cells of the cell-cycle data set are visualized in Circos [58]. The max function is configured for our method. The banded ideograms are mouse chromosomes (1-19, X and Y) and the black lines between them are interchromosomal interactions and the ends of these lines correspond to identified regions in chromosomes. Figure 2a shows the results of single cells of G1 phase; Fig. 2b shows the results of single cells of Early-S phase; Fig. 2c shows the results of single cells of Mid-S phase; and Fig. 2d shows the results of single cells of Late-S phase.

Among all four Circos plots, there is an apparent common **hub** in chromosome 11 (between 3Mb and 3.5Mb) whose interchromosomal interactions are highly enriched. The finding of this hub is corroborated by previous research with bulk Hi-C experiments to study interchromosomal contact networks in mammalian genomes [55]. They also discovered this hub in the mouse genome. Our finding confirms the hub’s existence at single-cell level and rules out the possibility that its existence is solely contributed by very few cells with a large amount of interchromosomal interactions in the region. In addition, these four Circos plots are similar but not exactly the same, which means single cells of different cell phases share some interchromosomal interactions but also have some variabilities on interchromosomal interactions.

**Fig. 3:**
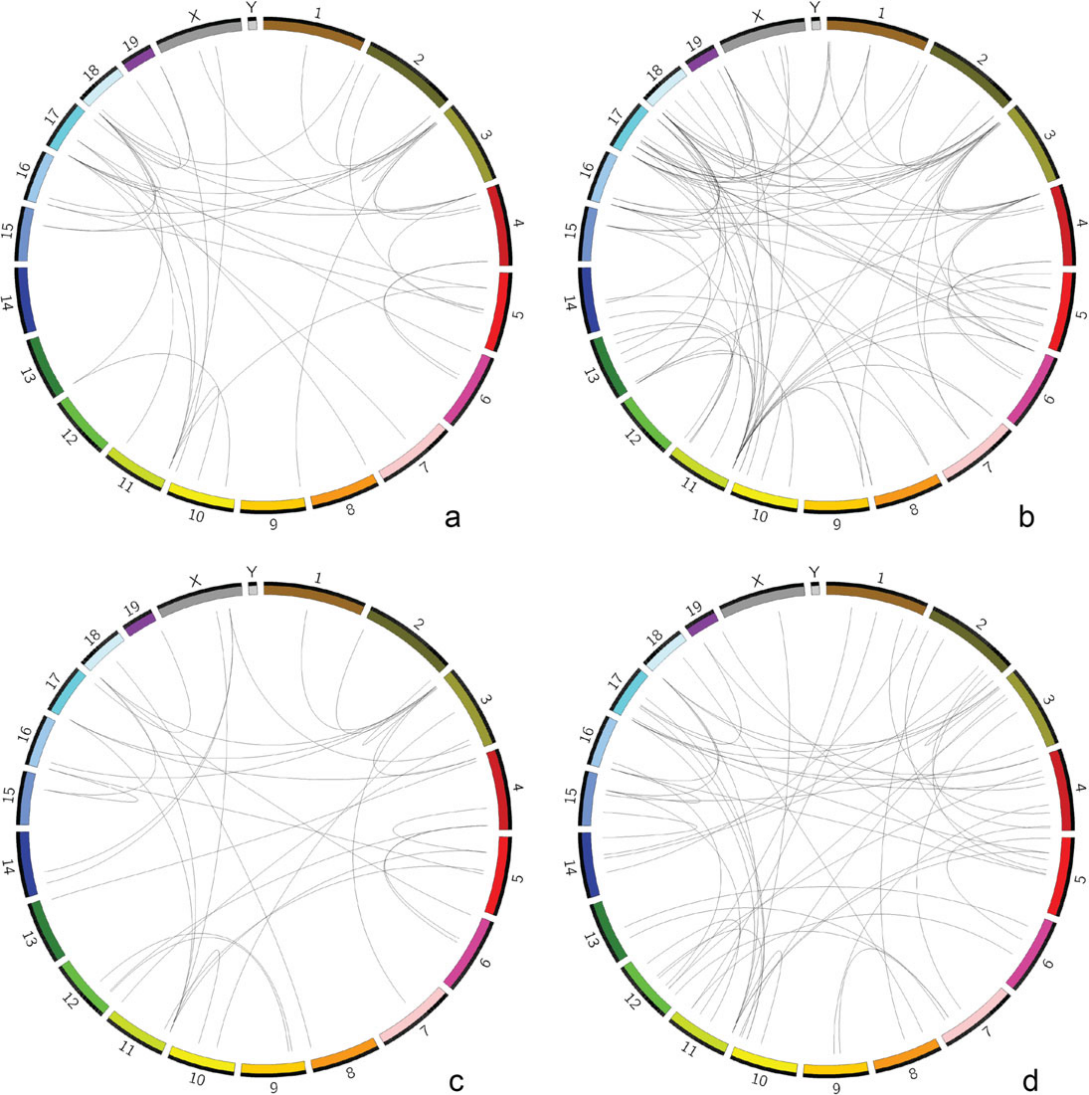
Identified regions of the oocyte-to-zygote data set. Visualizing genome-wide identified regions and their interchromosomal interactions of the oocyte-to-zygote data set with an adjusted *p*-value cutoff of 0.05 in Circos plots. **a** single oocytes labeled as NSN; **b** single oocytes labeled as SN; **c** maternal nuclei from zygotes; **d** paternal nuclei from zygotes

In Fig. 3, identified regions associated with statistically frequent interchromosomal interactions among single cells/nuclei of the oocyte-to-zygote data set are visualized. Figure 3a shows the results of single oocytes labeled as NSN; Fig. 3b shows the results of single oocytes labeled as SN; Fig. 3c shows the results of single maternal nuclei from zygotes; and Fig. 3d shows the results of single paternal nuclei from zygotes. Our tool reports much fewer regions on this data set and there is no hub. The absence of the hub may be partly because of cell discrepancies on cell types and cell cycles. To be more specific, in the second research, oocytes and maternal/paternal nuclei from zygotes only contain a single set of chromosomes. However, for the chromosome 11 from 3Mb to 3.5Mb, there are comparatively more interchromosomal interactions among all four Circos plots. Additionally, a similar interchromosomal interaction pattern is observed: there are some shared interchromosomal interactions but there are also some variabilities at single-cell resolution.

#### Pairwise comparisons of identified regions

For the cell-cycle data set, we compare the identified regions from single cells of different phases and examine the similarity and dissimilarity. In Table 1, single cells of different phases share a lot of common regions. There are some unique regions in each phased single cells. All pairs have more common regions than unique regions except the comparison between Early-S and Mid-S. Because the number of common regions is limited by the identified regions from single cells at Mid-S phase and single cells at Early-S phase report the most identified regions.

**Table 1:** Pairwise comparisons of the cell-cycle data set

Comparison	Common	Unique in former	Unique in latter
G1 VS Early-S	757	219	569
G1 VS Mid-S	526	450	198
G1 VS Late-S	708	268	335
Early-S VS Mid-S	595	731	129
Early-S VS Late-S	767	559	276
Mid-S VS Late-S	597	127	446

We also compare the identified regions from single cells of the oocyte-to-zygote data set. In Table 2, single cells of different conditions share some regions and there are more unique regions than common regions. This phenomenon seems inconsistent with what we have observed in the cell-cycle data set. But it does make sense and reflects the different types of single cells/nuclei used in their experiments. When identified regions from oocytes labeled NSN are compared with the ones from other cells/nuclei, the oocytes labeled SN share the most common regions because both of them are the same type of cells and their common regions are limited by the identified regions from oocytes labeled NSN; single maternal nuclei share more regions than single paternal nuclei because oocytes and single maternal nuclei are both from females while single paternal nuclei are from males. The same reason can also be applied to explain why oocytes labeled SN share more common regions with single maternal nuclei than single paternal nuclei. At last, single maternal nuclei and single paternal nuclei share the fewest common regions because some are from females and the others are from males.

**Table 2:** Pairwise comparisons of the oocyte-to-zygote data set

Comparison	Common	Unique in former	Unique in latter
NSN VS SN	**35**	2	49
NSN VS maternal	18	19	15
NSN VS paternal	15	22	36
SN VS maternal	**21**	63	12
SN VS paternal	19	65	32
maternal VS paternal	**13**	20	38

#### Enrichment analysis of identified regions

To improve the interpretation of identified regions, we carry out enrichment analysis of identified regions with genomic features, which are available in the cell-cycle data set. As there are too many identified regions in the data set, we select top ranked regions/nodes according to the numbers of statistically frequent unweighted edges with a cutoff (≥3 except ≥4 for single cells at Early-S phase because there are too many top regions). Therefore we obtain 16 regions for single cells at G1 phase, 37 regions for single cells at Early-S phase, 34 regions for single cells at Mid-S phase and 47 regions for single cells at Late-S phase. Genomic features of mESC cell line are downloaded from this paper [59] and they are CTCF binding sites, enhancer sites, H3K4me3 peaks, H3K27ac peaks and Pol II peaks.

For the above selected regions of each phase, the numbers of genomic features are counted respectively. Then we ranomly select the same number of regions and count the numbers of genomic features falling into these randomly selected regions respectively. We carry out this randomization strategy 50,000 times and therefore we obtain empirical background samples for each genomic feature. We calculate the z-score for each genomic feature. In Table 3, most of genomic features are enriched (≥1.97, which corresponds to 0.05 in *p*-value) except enhancer. What is more important, for single cells at Early-S phase, all the genomic features are highly enriched. (When ≥3 is used as the cutoff, the results become more enriched.) H3K4me3 and H3K27ac are active gene transcirption marks. Pol II plays very important roles in gene transcription. An enhancer increases the likelihood of gene transcription. CTCF plays important rols in chromatin structure and insolates the spread of heterochromatin. Early-S phase corresponds to the commencement of DNA replication. These genomic features seems working coordinately to facilitate the initialization of DNA replication.

**Table 3:** Identified Regions’ Enrichment Analysis of the cell-cycle data set

Input	CTCF	enhancer	H3K4me3	H3K27ac	Pol II
G1	2.82	1.05	1.75	2.63	2.48
Early-S	**10.86**	**9.81**	**12.48**	**12.05**	**12.58**
Mid-S	2.81	1.48	3.08	2.74	3.64
Late-S	3.37	1.74	4.33	4.36	5.05

### Flexibility of configurations

To make our tool flexible to accommodate different scHi-C experiments, we support different configurations, which include sliding windows for region diversity, edge probability functions for adjustability of identified regions and different bin sizes.

#### Configuration of sliding windows

By default, our tool divides chromosomes into bins from the first bases of chromosomes to the last ones, which limits the starting and ending positions of regions. To overcome this limitation, our tool supports a sliding window strategy by moving bins toward the last bases certain bases (e.g. 100kb). It lets users decide where their regions’ starting and ending positions through a parameter. In Table 4, we adopt four sliding windows of sizes of 100kb, 200kb, 300kb and 400kb and compare the identified regions with the ones by default (no sliding window). If identified regions mediated by some interchromosomal interactions from the no sliding window condition overlap with identified regions from a sliding window condition at both ends, we treat these regions as common identified regions; otherwise they are different. Therefore, we can calculate the common identified regions between no sliding window and sliding windows. In Table 4, we conclude that most identifed regions between no sliding window and sliding windows are common because some shared interchromosomal interactions fall into these regions. But as these common regions’ starting and ending positions are different, our tool diversifies the identified regions to users. What is more interesting is the single cells at Early-S phase share the fewest identified regions between no sliding window and sliding windows of different sizes. As DNA synthesis commences at Early-S phase, interchromosomal interactions may vary or involve in DNA synthesis initialization activites more at this phase than other phases. In Table 5 of the oocyte-to-zygote data set, we can reach the same conclusion that most identified regions are common between no sliding window and sliding windows of different sizes and meanwhile there are some different regions.

**Table 4:** Overlapping identified regions of the cell-cycle data set with no sliding window and sliding windows of different sizes

Input Data	100kb	200kb	300kb	400kb
G1	92.11%	92.01%	92.01%	95.49%
Early-S	85.52%	86.05%	86.73%	89.22%
Mid-S	90.33%	89.92%	91.16%	93.65%
Late-S	93.19%	91.08%	91.66%	94.44%

**Table 5:** Overlapping identified regions of the oocyte-to-zygote data set with no sliding window and sliding windows of different sizes

Input Data	100kb	200kb	300kb	400kb
oocyte NSN	100%	92.01%	92.01%	95.49%
oocyte SN	86.90%	89.29%	89.29%	92.86%
pronucleus maternal	93.94%	93.94%	90.91%	100%
pronucleus paternal	94.12%	90.20%	90.20%	92.16%

#### Configuration of edge probability functions

Our proposed tool supports three functions, *max*, *mean* and *min*, to estimate the probability of an edge between two nodes of different chromosomes, therefore improving adjustability of identified regions. In Table 6 of the cell-cycle data set and Table 7 of the oocyte-to-zygote data set, our tool configured with the *max* function identifies the fewest regions; our tool configured with the *min* function identifies the most regions and our tool configured with the *mean* funciton falls between them. This is because if we fix other variables except *p* in Eq. 1, a large *p* entails a large *p*-*value* and a small *p* entails a small *p*-*value*. As we have explained in the second to last paragrpah of Method, users can select these functions according to the sizes of genomes and sequencing depths used in their experiments. Therefore, our proposed tool provides adaptability of identified regions.

**Table 6:** Number of identified regions of the cell-cycle data set with edge probability functions

Input Data	*max*	*mean*	*min*
G1	976	1651	2133
Early-S	1326	2579	7714
Mid-S	724	1833	2991
Late-S	1043	1999	6058

**Table 7:** Number of identified regions of the oocyte-to-zygote data set with edge probability functions

Input Data	*max*	*mean*	*min*
oocyte NSN	37	79	199
occyte SN	84	229	1846
pronucleus maternal	33	50	268
pronucleus paternal	51	51	274

#### Configuration of bin sizes

**Fig. 4:**
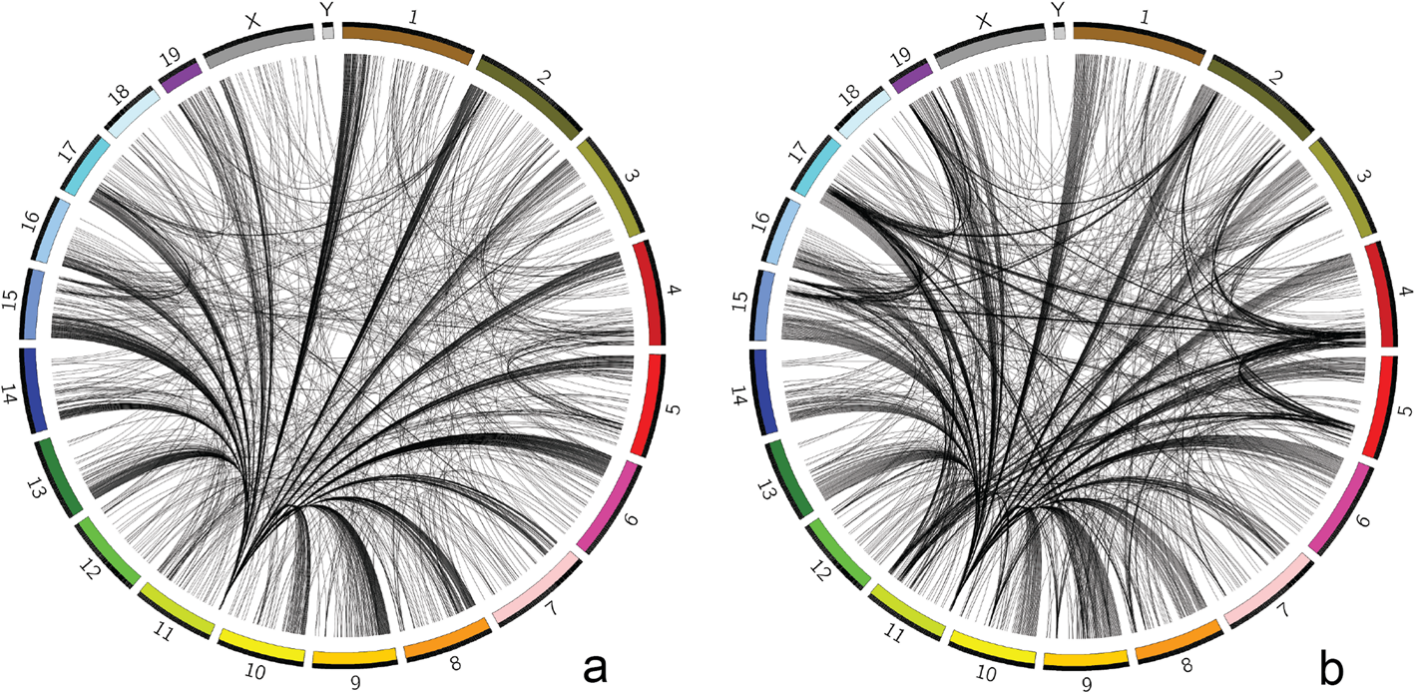
Comparing identified regions of Early-S phased single cells with different bin sizes. **a** bin_size=500kb **b** bin_size=1Mb

Finally, our tool also supports different bin sizes. As scHi-C experiments have low read coverages and scarce interchromosomal interactions, we need to use large bin sizes to accumulate sufficient interchromosomal interactions in a bin. We run our tool with bin_size=1Mb on the two data sets and compare the identified regions with the ones of bin_size=500kb. We find that the identified regions of bin_size=500kb and bin_size=1Mb are quite similar for most single cells except the Early-S phased single cells in the cell-cycle data set. In Fig. 4b of bin_size=1Mb, the hub of the chromosome 11 at 3Mb becomes less obvious as it is overshadowed by enrichment of other interchromosomal interactions because of the increased bin size and single cells of this particular cell phase. Therefore, different bin sizes may affect the identified regions.

## Conclusion

In this paper, we introduce a computational method to identify regions associated with statistically frequent interchromosomal interactions at single-cell resolution and implement it as an open source tool, which is the first serving the purpose to the best of our knowledge. Its workflow includes network construction, binomial statistical measurement calculation and region selection. We demonstrate its usability on two existing scHi-C data. On the cell-cycle data set, the tool discovers a hub in the mouse chromosome 11 from 3Mb to 3.5Mb, which is endorsed by a previous study on interchromosomal contact networks with bulk Hi-C experiments. On the oocyte-to-zygote data set, there is no apparent hub at the region, but comparatively interchromosomal interactions are enriched. Identified regions’ pairwise comparisons show that our method identifies common regions between different data sets and also reflects the true dissimilarity such as different cell types. Identified regions’ enrichment analysis helps improve the interpretation of top ranked identified regions and these genomic features are highly enriched for single cells at Early-S phase, which implies our top ranked regions may be functionally important. We also exhibit our proposed tool’s flexibility on configurations, which support sliding windows for diverse regions, edge probability functions for adjustable regions and different bin sizes. Overall, it will be a useful tool for analyzing scHi-C interchromosomal interactions.

Due to low sequencing depths of scHi-C experiments and the paucity of interchromosomal interactions, identifying high resolution regions of several kilobases (e.g. 8kb) is extremely difficult. Our tool can run with this resolution but due to the limitation of scHi-C data, it can’t identify any regions passing the statistical tests. We will try to mitigate this problem by imputing high-resolution interchromosomal interactions with data of other experiments such as interchromosomal interactions from bulk Hi-C experiments. In addition, further research is needed to improve the signal-to-noise ratio for scHi-C experiments.

## Data Availability

For the implementaion details of our tool, please check out it at https://github.com/bignetworks2019/Inter-chromosomal-interactions. Currently it supports the following genomes, mm9, mm10, hg18 and hg19. It can be easily extended to other organisms.

## Abbreviations

Hi-C: chromosome conformation capture with high-throughput sequencing
TAD: topologically associating domain
scHi-C: single-cell chromosome conformation capture with high-throughput sequencing
kb: kilobases
Mb: megabases
mESC: mouse embryonic stem cell
NSN: non-surrounded nucleolus
SN: surrounded nucleolus
